# Magnetic resonance imaging assessment of the changes of cardiac and hepatic iron load in thalassemia patients before and after hematopoietic stem cell transplantation

**DOI:** 10.1038/s41598-023-46524-y

**Published:** 2023-11-10

**Authors:** Fengming Xu, Da Li, Cheng Tang, Bumin Liang, Kaiming Guan, Rongrong Liu, Peng Peng

**Affiliations:** 1 Department of Radiology, The First Affiliated Hospital of Guangxi Medical University, Nanning, 530021 Guangxi Zhuang Autonomous Region People’s Republic of China; 2 Department of Gastrointestinal Surgery, The First Affiliated Hospital of Guangxi Medical University, Nanning, 530021, Guangxi Zhuang Autonomous Region People’s Republic of China; 3 NHC Key Laboratory of Thalassemia Medicine (Guangxi Medical University), Nanning, Guangxi Zhuang Autonomous Region People’s Republic of China; 4 School of International Education, Guangxi Medical University, Nanning, 530021 Guangxi Zhuang Autonomous Region People’s Republic of China; 5 Department of Haematology, The First Affiliated Hospital of Guangxi Medical University, Nanning, 530021 Guangxi Zhuang Autonomous Region People’s Republic of China

**Keywords:** Haematological diseases, Haematopoietic stem cells

## Abstract

To investigate the value of T_2_^*^ technique on 3.0 T magnetic resonance imaging (MRI) in evaluating the changes of cardiac and hepatic iron load before and after hematopoietic stem cell transplantation (HSCT) in patients with thalassemia (TM), the 141 TM patients were divided into 6 group for subgroup analysis: 6, 12, 18, 24 and > 24 months group, according to the postoperative interval. The T_2_^*^ values of heart and liver (H-T_2_^*^, L-T_2_^*^) were quantified in TM patients before and after HSCT using 3.0 T MRI T_2_^*^ technology, and the corresponding serum ferritin (SF) was collected at the same time, and the changes of the three before and after HSCT were compared. The overall H-T_2_^*^ (*P* = 0.001) and L-T_2_^*^ (*P* = 0.041) of patients after HSCT were higher than those before HSCT (mean relative changes = 19.63%, 7.19%). The H-T_2_^*^ (*P* < 0.001) and L-T_2_^*^ (P < 0.001) > 24 months after HSCT were significantly higher than those before HSCT (mean relative changes = 69.19%, 93.73%). The SF of 6 months (*P* < 0.001), 12 months (*P* = 0.008), 18 months (*P* = 0.002) and > 24 months (*P* = 0.001) were significantly higher than those before HSCT (mean relative changes = 57.93%, 73.84%, 128.51%, 85.47%). There was no significant improvement in cardiac and liver iron content in TM patients within 24 months after HSCT, while the reduction of cardiac and liver iron content in patients is obvious when > 24 months after HSCT.

## Introduction

Thalassemia (TM) is an inherited autosomal recessive hematologic disorder caused by mutations in the globin gene or its regulatory region. This results in a reduced rate of synthesis of the globin chains that make up hemoglobin, and the patient's body cannot make adequate amounts of normal hemoglobin. The traditional treatment is to correct the hemoglobin status through regular blood transfusions to relieve anemia. However, traditional methods cannot fundamentally cure the disease^1^. Currently, the only method with curative potential is hematopoietic stem cell transplantation (HSCT)^2^. Over the past 2 decades, with advances in strategies to manage transplant-related complications and the reduction of toxicity associated with preparation protocols, post-transplant outcomes of transfusion-dependent TM have improved significantly. Studies have shown that the early HSCT, before the accumulation of TM-related organ dysfunction, is associated with reduced transplant-related toxicity. When HSCT is performed with an HLA-matched sibling donor before the age of 14 years, transplatment-related mortality is less than 10%, and is further reduced to less than 5% if transplantation is performed before the age of 5 years^3^.

However, after HSCT, blood reconstitution (implantation to initial recovery, and then to complete recovery) requires a certain period of time^4^, during which some patients still require regular blood transfusion therapy. Regular blood transfusions can lead to the accumulation of iron in the body's organs, which can lead to multiple organ disease and premature death unless regular iron chelation therapy is administered^5–9^. Therefore, whether it is traditional blood transfusion therapy or after HSCT, it is necessary to regularly monitor the iron content of patients' organs.

The T_2_^*^, R_2_^*^ (1000/T_2_^*^) technique based on Magnetic resonance imaging (MRI) gradient recalled echo (GRE) imaging sequences has been identified as a non-invasive standard for quantifying tissue iron content^10,11^. Due to the non-invasiveness, reproducibility and high safety of this technology, long-term and regular monitoring of the iron content of patients' organs can be realized. Many studies have used 1.5 T MRI to explore the relationship between T_2_^*^, R_2_^*^ and iron load in organs, which not only determined the negative correlation trend between T_2_^*^ and organ iron load, but also constructed the corresponding calibration curve equation to calculate the corresponding iron concentration^12–17^. At the same time, a small number of studies have used 3.0 T MRI to explore the relationship between T_2_^*^, R_2_^*^ and iron content in organs^18,19^. However, there are few studies on the quantitative assessment of visceral iron content in TM patients after HSCT by this technique.

The aim of this study was to quantitatively assess the content of cardiac and hepatic iron in patients with TM after HSCT by 3.0 T MRI T_2_^*^ technology, the changes of cardiac and hepatic iron load in TM patients after HSCT is investigated.

## Materials and methods

### Ethics statement

This study was conducted in accordance with the principles of the Declaration of Helsinki and approved by the Ethics Committee of the First Affiliated Hospital of Guangxi Medical University (NO.2022-E358-01). And since this study was retrospective, verbal informed consent notification was given to all subjects.

### Study design

**Figure 1:**
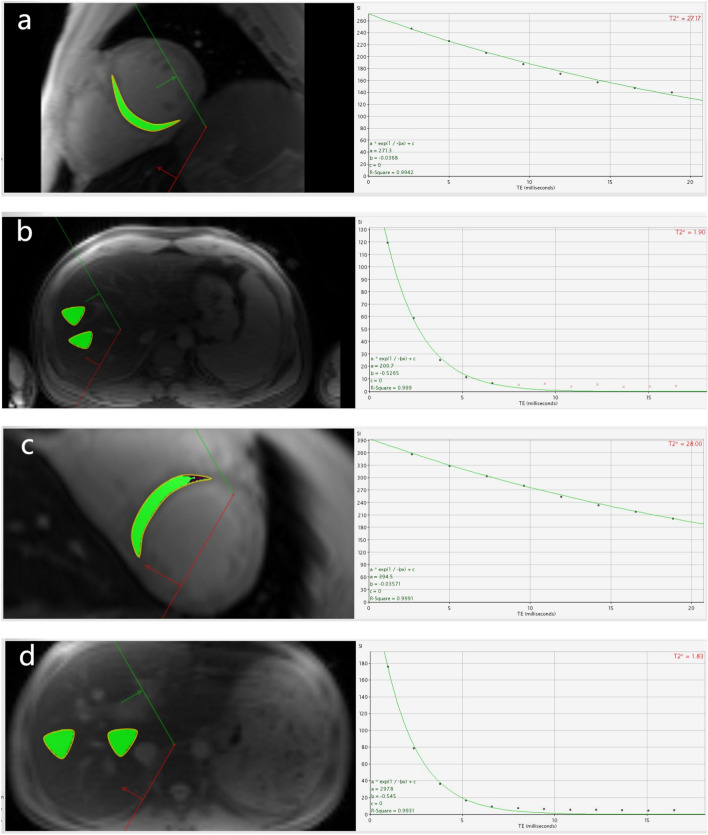
(**a**,**b**) β-TM patient, 25-year-old male, preoperative examination of HSCT. CMRTools in FIG. 1a shows that the average value of H-T_2_^*^ is 27.17, R^2^ (goodness of fit) is 0.9942, and 8 echo SI are included. CMRTools in FIG. 1b shows that the average value of L-T_2_^*^ is 1.90, R^2^ is 0.999, the SI of the first five echoes are included, and the cross represents the SI of the discarded deviation from the fitted curve. (**c**,**d**) β-TM patient, a 14-year-old male, was examined after HSCT. CMRTools in FIG. 1c shows that the average value of H-T_2_^*^ is 28.00, R^2^ is 0.9991, and 8 echo SI are included. CMRTools in (**d**) shows that the average L-T_2_^*^ is 1.83, R^2^ is 0.9931, and 12 echo SI are included.

The T_2_^*^ imaging and clinical data of 218 TM patients who had been treated with HSCT in our center from January 2015 to June 2022 were retrospectively analyzed. All T_2_^*^ image data were measured by CMRtools (CMRtools/Thalassemia Tools 2014, Cardiovascular Imaging Solutions, London, UK). Measurement process: Image data was exported through the PACS system and imported into a personal computer with installed CMRtools software. The Thalassaemia Assessment function of CMRtools was used. For the heart, the largest possible area of the ventricular septum Region in the first echo image (the software automatically delineated the remaining echo image) was delineated as the Region of Interest (ROI). For the liver, two ROIs of about 2–4 cm^2^ in size were drawn in the right lobe of the liver in the first echo image, avoiding the intrahepatic vessels and bile ducts seen by the naked eye. The delineated ROI and fitted T_2_^*^ values appeared in the post-processing software. The truncation method^19^ was used to round off the signal intensity (SI) values that deviated from the fitted curve one by one from back to front, and when the coefficient of determination (R^2^) value was ≥ 0.98, the T_2_^*^ values were recorded (Fig. 1). According to Garbowski curve^16^ and T_2_^*^ classification, cardiac T_2_^*^ was divided into severe group (< 5.7 ms), moderate group (5.7–7.2 ms), mild group (7.2–10.5 ms) and normal group (> 10.5 ms). Liver T_2_^*^ was divided into severe group (< 1.59 ms), moderate group (1.59–2.17 ms), mild group (2.17–3.78 ms) and normal group (> 3.78 ms).

All clinical data were collected through the HIS system of our institution, including serum ferritin within the matching time of MRI scan (< 1 week).

### Inclusion and exclusion criteria

Inclusion criteria: (1) patients diagnosed with TM by genetic diagnosis. (2) Any type of HSCT, including bone marrow transplantation (BMT), peripheral blood cell transplantation (PBCT), or (umbilical cord blood transplantation (UCBT). Cardiac and liver GRE sequence MRI examinations were performed before and after HSCT. (3) Treatment by regular or irregular blood transfusion. (4) Regular or irregular iron removal treatment. (5) Age ≥ 4 years. The exclusion criteria: (1) GRE image data artifacts were large and did not meet the measurement requirements. (2) Patients combined with other chronic liver diseases or tumor diseases. (3) Failure of HSCT or postoperative complications.

### Magnetic resonance imaging (MRI) protocol

Siemens 3 T MRI scanner (Prisma, Siemens Healthcare, Erlangen, Germany) was used. Abdominal coils and multi-echo GRE sequences were used. (1) Cardiac scan: 8 echo bright blood sequence of ECG gated single breath hold was used, and the scanning layer with better ventricular septum was selected in the short-axis view. TR 138 ms, TE were 2.68, 4.99, 7.3, 9.61, 11.92, 14.23, 16.54, 18.85 ms, turning Angle was 20°, matrix = 128 × 256, FOV = 226 mm × 400 mm, layer thickness was 10 mm, scanning time was about 12 s. (2) Liver scan: 12 echo sequences were examined in a single breath hold, and large blood vessels were avoided during layer selection. TR 200 ms, TE 0.97, 2.38, 3.79, 5.20, 6.61, 8.02, 9.43, 10.84, 12.25, 13.66, 15.07, 16.48 ms, turning Angle was 20°, matrix 64 × 128, FOV 200 mm × 400 mm, The layer thickness was 10 mm, and the scanning time was about 10 s.

### Statistical analysis

SPSS 26.0 statistical software package was used to analyze the total H-T_2_^*^, L-T_2_^*^, and SF before and after HSCT. The patients were divided into 6-month group (< 6 months), 12-month group (6–12 months), 18-month group (12–18 months), 24-month group (18–24 months) and > 24 months group according to the postoperative interval. Statistical analysis was performed according to the test level α = 0.05.

Using intraclass correlation coefficient (ICC), liver T_2_^*^ image data of 28 patients were randomly selected for measurement to evaluate the consistency of measurement results within and between observers. Observers A and B (radiologists with 5 years of experience in abdominal and cardiothoracic radiology) each took two measurements of liver T_2_^*^ images in these 28 patients. Intra-observer ICC was calculated by comparing T_2_^*^ values measured twice by each observer. Interobserver ICC was calculated by comparing the mean values measured by observers A and B. Measurement tasks for all patients were averaged and randomly assigned to observers A and B.

Kolmogorov–smirnov test (K–S) was used to test normality for sample size (n) > 50. 3 < n < 50 were tested for normality using the Shapiro–Wilk test (S–K). Enumeration data are described in percentage (%). The measurement data that conform to the normal distribution are described by the mean ± standard deviation ($\overline{x} \pm s$). Paired t-test (matched samples t-test) was used for difference analysis; Pearson correlation coefficient was used to explore correlation. Measurement data that do not conform to normal distribution are described by median (M) and interquartile range (P_25%_–P_75%_). Mann–Whitney U test was used for difference analysis; Spearman correlation coefficient was used to explore correlation.

### Ethics approval and informed consent

The study was approved by the Ethics Committee of the First Affiliated Hospital of Guangxi Medical University (NO.2022-E358-01). Since this study was retrospective, the subjects were exempted from their informed consent.

## Results

A total of 141 patients were included, including 86 males (60.99%) and 55 females (39.01%), with an age of 9.56 ± 2.84 (Mean ± standard deviation). Among the 141 patients, 131 (92.91%) were severe β-TM, and 10 (7.09%) were severe α-TM. Twenty-nine patients (20.57%) had undergone splenectomy before HSCT.

### Analysis of normality

**Figure 2:**
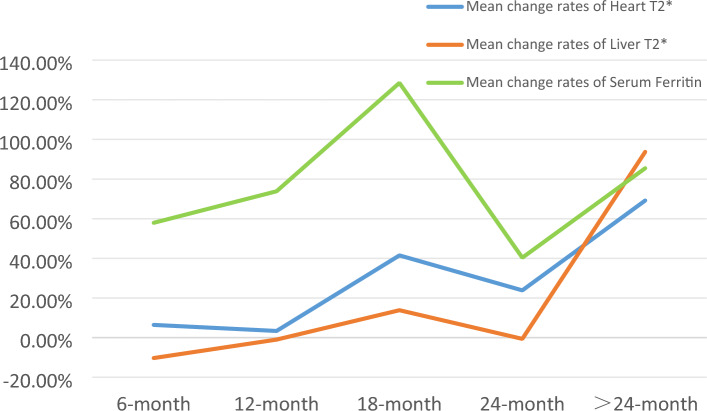
A line plot based on the mean change rates (%) over time of the change in Heart T_2_^*^ (ms), Liver T_2_^*^ (ms), and Serum Ferritin (μg/l) before and after HSCT in TM patients.

The H-T_2_^*^, L-T_2_^*^ and SF statistics are described in Table 1. After the normality test, the normal distribution (*P* > 0.05) was seen in total preoperative H-T_2_^*^, total postoperative H-T_2_^*^, preoperative and postoperative H-T_2_^*^ in the 6 month group, preoperative and postoperative H-T_2_^*^ in the 12 month group, postoperative H-T_2_^*^ in the 18 month group, postoperative L-T_2_^*^ in the 24 month group, preoperative and postoperative H-T_2_^*^ in the 24 month group, preoperative and postoperative SF in the 24 month group, and preoperative and postoperative H-T_2_^*^ in the > 24 month group. Figure 2 shows the mean change rates (%) over time of the change in heart, liver T2 * and serum ferritin: H-T_2_^*^ and L-T_2_^*^ show a significant upward trend with time while SF showed a relatively large fluctuation.

**Table 1:** Statistical description indexes of H-T_2_ * (ms), L-T_2_^*^ (ms), and SF (μg/l) before and after HSCT in TM patients.

Variable	Group before (1)/after (2) HSCT	Frequency	Minimum value–Max value	Median	Interquartile range (P_25%_–P_75%_)	Mean ± standard deviation ($\overline{x}$ ± *s*)	Mean change rate (%)
H-T_2_*	Overall 1	141	2.65–39.73	15.39	10.77–22.18	16.68 ± 8.55	19.63
	Overall 2	141	2.15–48.66	19.36	12.12–26.50	19.95 ± 10.36	
	6-month 1	72	3.31–37.65	17.23	10.85–22.87	17.56 ± 8.49	6.43
	6-month 2	72	3.84–42.79	18.07	11.57–24.72	18.69 ± 9.00	
	12-month 1	24	3.9–35.31	16.57	10.01–20.81	16.18 ± 8.17	3.37
	12-month 2	24	4.35–34.65	14.87	8.43–22.21	16.72 ± 9.07	
	18-month 1	14	10.2–39.73	12.78	10.77–12.78	17.27 ± 9.31	41.49
	18-month 2	14	6.55–48.66	26.56	13.09–34.46	24.44 ± 12.79	
	24-month 1	10	2.65–31.08	14.45	11.32–23.33	16.60 ± 9.30	23.85
	24-month 2	10	2.15–30.96	24.46	15.68–25.45	20.56 ± 9.23	
	> 24 months 1	21	2.65–31.18	13.21	7.85–21.21	14.59 ± 8.68	69.19
	> 24 months 2	21	4.32–48.66	26.71	14.40–33.30	24.69 ± 12.94	
L-T_2_*	Overall 1	141	0.68–11.05	1.16	0.91–1.80	1.74 ± 1.67	7.19
	Overall 2	141	0.72–6.99	1.24	0.99–2.23	1.86 ± 1.35	
	6-month 1	72	0.70–11.05	1.18	0.94–1.80	1.84 ± 1.90	− 10.30
	6-month 2	72	0.75–5.77	1.16	0.99–1.92	1.65 ± 1.17	
	12-month 1	24	0.76–5.68	1.12	0.92–1.68	1.52 ± 1.05	− 0.99
	12-month 2	24	0.86–5.53	1.26	1.00–1.78	1.51 ± 0.95	
	18-month 1	14	0.69–3.12	1.02	0.83–2.58	1.48 ± 0.93	13.84
	18-month 2	14	0.72–6.99	0.98	0.83–2.22	1.69 ± 1.65	
	24-month 1	10	0.79–8.98	1.46	0.96–2.77	2.44 ± 2.57	− 0.62
	24-month 2	10	0.86–4.78	0.96	0.79–1.85	2.42 ± 1.34	
	> 24 months 1	21	0.68–3.82	0.96	0.79–1.85	1.40 ± 0.93	93.73
	> 24 months 2	21	0.85–6.99	2.37	1.25–3.86	2.71 ± 1.69	
SF	Overall 1	141	295.00–14,724.50	4552	3278.76–4829.46	4529.55 ± 2535.92	57.14
	Overall 2	141	563.72–30,605.05	7515	4246.62–8302.25	7117.74 ± 4120.65	
	6-month 1	72	295.00–14,724.50	4552	3228.58–5620.09	4827.90 ± 2953.06	57.93
	6-month 2	72	1974.58–23,014.05	7718.73	4251.43–8489.25	7624.69 ± 4452.57	
	12-month 1	24	1661.11–14,463.08	4552	2895.79–6065.93	4925.36 ± 2583.75	73.84
	12-month 2	24	3061.42–26,000.08	6996.115	4268.37–9316.36	8562.19 ± 6245.18	
	18-month 1	14	390.03–4552	4242.92	3882.28–4552	3951.25 ± 1093.78	128.51
	18-month 2	14	563.72–30,605.05	6971	4887.96–7718.73	9028.83 ± 8410.68	
	24-month 1	10	1264.00–5894.69	4263.505	1678.86–4612.045	3660.10 ± 1615.46	40.34
	24-month 2	10	925.00–9275.10	4224.495	2879.81–8117.56	5136.63 ± 2911.15	
	> 24 months 1	21	795.16–6086.13	4552	2936.25–4552	4007.80 ± 1410.98	85.47
	> 24 months 2	21	1358.00–30,605.05	7636.95	4269.49–8011.62	7433.36 ± 5762.13	

H-T_2_* is Heart T_2_*; L-T_2_* is Liver T_2_*; SF is Serum Ferritin; HSCT is hematopoietic stem cell transplantation; TM is thalassemia.

### Difference analysis of H-T_2_^*^, L-T_2_^*^and SF before and after transplantation

**Figure 3:**
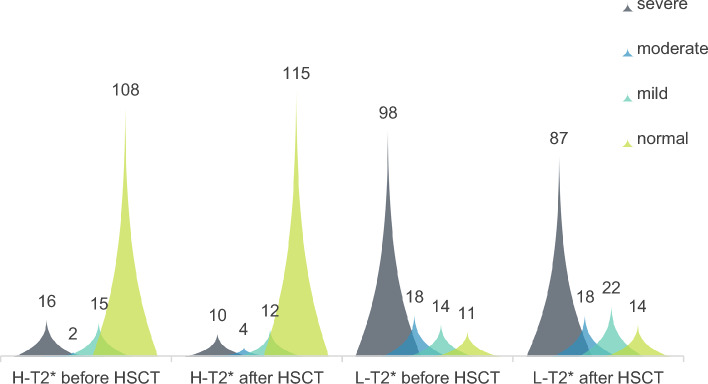
Overall distribution of Heart T_2_^*^ and liver T_2_^*^ grades before and after HSCT (the ordinate indicates the number of frequencies).

**Figure 4:**
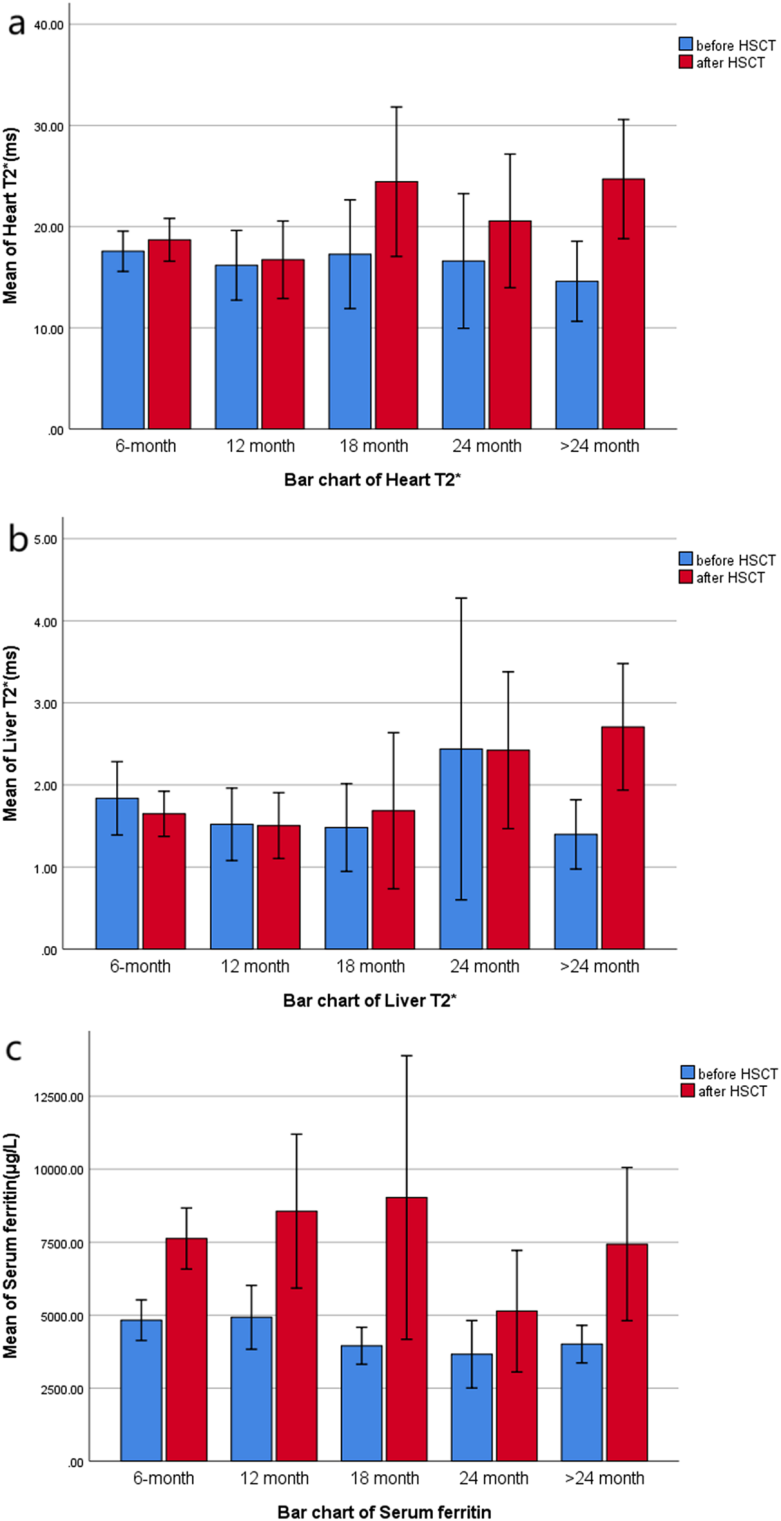
Bar graphs of heart T_2_^*^, liver T_2_^*^ and serum ferritin based on means.

Figure 3 cluster diagram shows the distribution of H-T_2_^*^ and L-T_2_^*^ grades before and after HSCT. After T_2_^*^ grading, the number of cases in severe and moderate liver iron concentration after HSCT decreased (preoperative n = 116, 82.27%; Postoperative n = 105, 74.47%), the number of cases in mild and normal groups increased (preoperative n = 25, 17.73%; Postoperative n = 36, 25.53%). After HSCT, the number of patients with severe and moderate cardiac iron concentration decreased (preoperative n = 18, 12.77%; postoperative n = 14, 9.93%), while the number of mild and normal groups increased (preoperative n = 123, 87.23%; postoperative n = 127, 90.07%). Figure 4a shows that H-T_2_^*^ in TM patients increased from 18 months after HSCT (Significant reduction in cardiac iron load); Fig. 4b shows that L-T_2_^*^ in TM patients increased from 24 months after HSCT (Significant reduction in hepatic iron load).

H-T_2_^*^: The overall H-T_2_^*^(*t* = − 3.510, *P* = 0.001) and the postoperative H-T_2_^*^(*t* = − 4.300, *P* < 0.001) of the > 24 months group were statistically different from the corresponding preoperative H-T_2_^*^. The mean value ($\overline{x} \pm s$) of H-T_2_^*^ increased significantly in the > 24 months group (preoperative = 14.59, range 2.65–31.18; Postoperative = 24.69, range 4.32–48.66). It is suggested that H-T_2_^*^ significantly increased after the postoperative interval of more than 24 months, that is, myocardial iron content significantly decreased. Postoperative H-T_2_^*^ (*t* = − 1.165, *P* = 0.248) in the 6-month group, postoperative H-T_2_^*^ (*t* = − 0.311, *P* = 0.758) in the 12-month group, postoperative H-T_2_^*^ (*Z* = − 1.328, *P* = 0.184) in the 18-month group and postoperative H-T_2_^*^ (*Z* = − 1.247, *P* = 0.203) in the 24-month group had no significant difference with the corresponding preoperative H-T_2_^*^.

L-T_2_^*^: The overall postoperative L-T_2_^*^ (*Z* = − 2.042, *P* = 0.041) and the postoperative L-T_2_^*^ (*Z* = − 3.945, *P* < 0.001) of the > 24 months group were statistically different from the corresponding preoperative L-T_2_^*^. The median (M) of L-T_2_^*^ increased significantly in the > 24 months group (preoperative M = 0.96, range 0.68–3.82, P_25%_–P_75%_ = 0.785–1.845; Postoperative M = 2.37, range 0.85–6.99, P_25%_–P_75%_ = 1.245–3.855). It is suggested that L-T_2_^*^ increased significantly after the postoperative time interval of more than 24 months, which means that the liver iron content decreased significantly. The L-_2_ * (*Z* = − 0.314, *P* = 0.753) in the 6-month group, L-T_2_^*^ (Z = − 0.015, *P* = 0.988) in the 12-month group, L-T_2_^*^ (*Z* = − 0.245, *P* = 0.0.807) in the 18-month group, and L-T_2_^*^ (*Z* = − 0.459, *P* = 0.646) in the 24-month group had no significant difference with the corresponding preoperative L-T_2_^*^.

SF: Overall SF (*Z* = − 7.911, *P* < 0.001), postoperative SF (*Z* = − 5.780, *P* < 0.001) in the 6-month group, postoperative SF (*Z* = − 2.658, *P* = 0.008) in the 12-month group, postoperative SF (*Z* = − 3.110, *P* = 0.002) in the 18-month group and > 24 months group postoperative SF (*Z* = − 3.251, *P* = 0.001) were statistically different from the corresponding preoperative SF. The median SF (M) of the 6, 12, 18 and > 24 months groups was significantly lower (preoperative total M = 4552, range 295.00–14,724.50, P_25%_–P_75%_ = 3278.76–4829.46; Postoperative M = 7515, range 563.72–30,605.05, P_25%_–P_75%_ = 4246.62–8302.25). It suggested that SF changed greatly after HSCT, and SF increased in most time periods after HSCT. There was no significant difference in postoperative SF (*t* = − 1.890, *P* = 0.091) between the 24-month group and preoperative SF.

### Correlation analysis of H-T_2_^*^, L-T_2_^*^, and SF before and after transplantation

**Figure 5:**
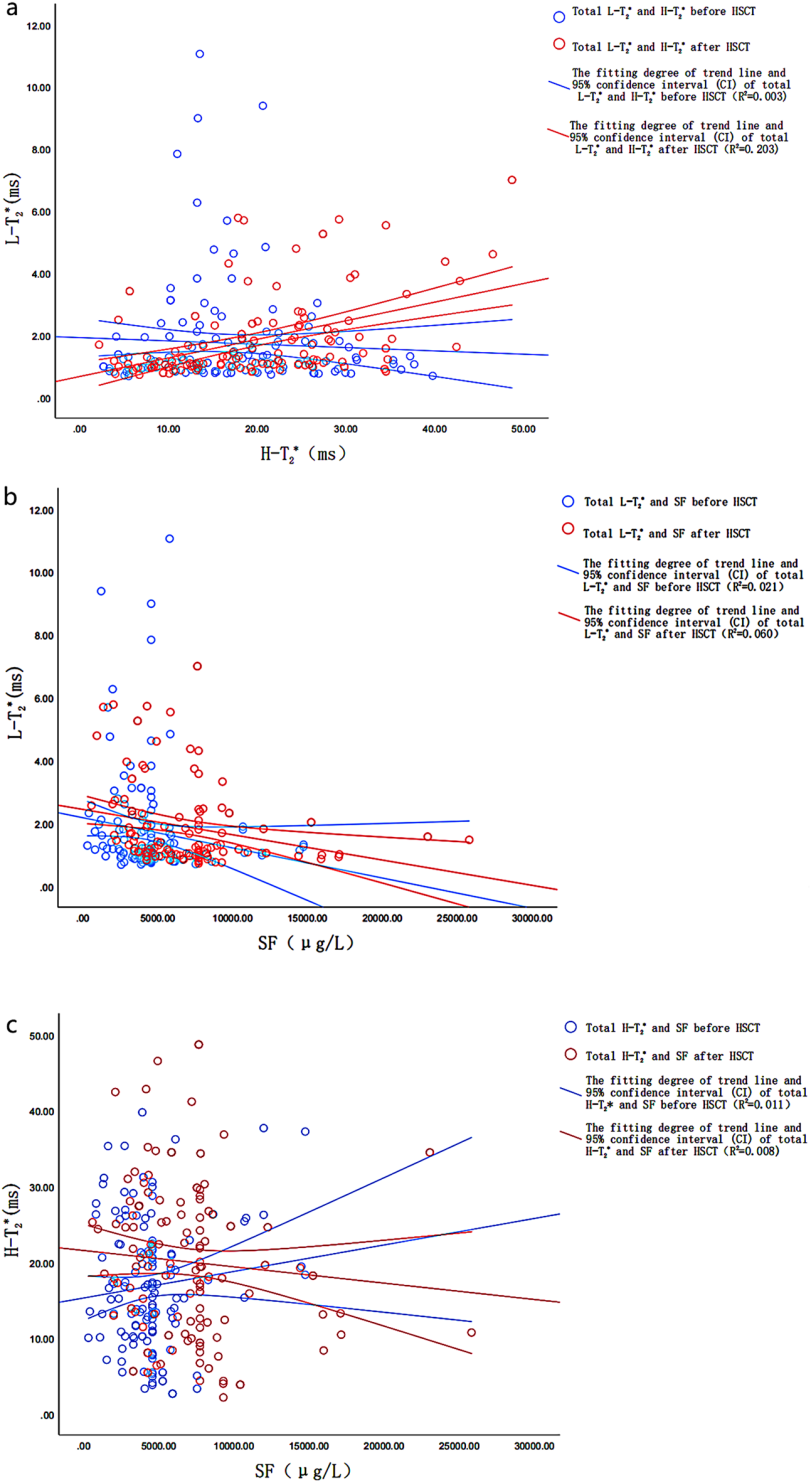
Scatter plot (**a**–**c**) shows that the fitting degree of trend line and 95% confidence interval (CI) of total liver T_2_^*^, heart T_2_^*^ and serum ferritin before and after HSCT is very low, showing low correlation or no correlation.

Figure 5 shows the scatterplots of the relationship between overall H-T_2_^*^, L-T_2_^*^and SF before and after HSCT. Before transplantation, there was no correlation between total H-T_2_^*^ and total L-T_2_^*^ (*r* = 0.001, *P* = 0.991). There was no correlation between total H-T_2_^*^ and preoperative total SF (*r* = − 0.097, *P* = 0.255). There was a low negative correlation between L-T_2_^*^ and SF (*r* = − 0.222, *P* = 0.009). There was no correlation between H-T_2_^*^, L-T_2_^*^and SF in the 6-month group (*P* = 0.110, 0.996, 0.298). There was no correlation between H-T_2_^*^ and L-T_2_^*^, and between L-T_2_^*^ and SF in the 12-month group (*P* = 0.230, 0.762). There was a moderate negative correlation between H-T_2_^*^ and SF (*r* = − 0.441, *P* = 0.031). There was no correlation between preoperative H-T_2_^*^, L-T_2_^*^and SF in the 18-month group (*P* = 0.215, 0.286, 0.775). There was no correlation between H-T_2_^*^ and L-T_2_^*^, and between L-T_2_^*^ and SF in the 24-month group (*P* = 0.934, 0.238). There was a high negative correlation between H-T_2_^*^ and SF (*r* = − 0.804, *P* = 0.005). There was no correlation between H-T_2_^*^, L-T_2_^*^ and SF in the > 24 months group (*P* = 0.223, 0.960, 0.740).

After transplantation: There was a low positive correlation between total H-T_2_^*^ and total L-T_2_^*^(*r* = 0.382, *P* < 0.001). There was no correlation between total H-T_2_^*^ and total SF (*r* = − 0.097, *P* = 0.255). There was a low negative correlation between L-T_2_^*^ and SF (*r* = − 0.340, *P* < 0.001). In the 6-month group, there was a moderate positive correlation betweenH-T_2_^*^ and L-T_2_^*^ (*r* = 0.419, *P* < 0.001) and a low negative correlation between L-T_2_^*^ and SF (*r* = − 0.394, *P* = 0.001), while there was no correlation between H-T_2_^*^ and SF (*P* = 0.503). In the 12-month group, there was a high positive correlation between H-T_2_^*^ and L-T_2_^*^ (*r* = 0.704, *P* < 0.001). There was no correlation between H-T_2_^*^ and SF, and between L-T_2_^*^ and SF (*P* = 0.200, 0.278). There was no correlation between H-T_2_^*^, L-T_2_^*^ and SF in 18-month group (*P* = 0.706, 0.810, 0.701). In the 24-month group, there was no correlation between H-T_2_^*^ and L-T_2_^*^ (*P* = 0.403); there was a moderate negative correlation between H-T_2_^*^ and SF (*r* = − 0.673,* P* = 0.033); there was a high negative correlation between L-T_2_^*^ and SF (*r* = − 0.723, *P* = 0.018). There was no correlation between H-T_2_^*^, L-T_2_^*^and SF in the > 24 months group (*P* = 0.253, 0.424, 0.996).

### Red blood cell infusion and iron removal chelation therapy

A total of 115 patients' pre-HSCT red blood cell transfusion and iron chelation therapy were collected (some patients' data were missing). Different units of red blood cells were transfused in different TM patients (1U of red blood cells separated from 200 ml of whole blood) : 1U (n = 4, 3.5%), 1.25U (n = 2, 1.7%), 1.5U (n = 10, 8.7%), 1.75U (4, 3.5%), 2U (n = 72, 62.6%), 2.5U (n = 3, 2.6%), 3U (n = 11, 9.6%), 3.5U (3, 2.6%), 3.75U (n = 1, 0.9%), 4U (n = 4, 3.5%), 4.5U (n = 1, 0.9%). All the 115 patients received iron chelation therapy (deferiprone, deferoxamine, deferasirox): 15 cases (13%) received irregular chelation, 78 cases (67.8%) received regular chelation with single drug, 14 cases (12.2%) received regular chelation with combination of two drugs, 8 cases (7%) received regular chelation with combination of three drugs.

### Test of Consistency

Intra-observer ICC = 0.996 (95% CI 0.991–0.998) calculated based on 2 measurements of observer A, and *P* < 0.001. Intra-observer ICC = 0.999 (95% CI 0.999–1.000) calculated based on 2 measurements of observer B, and *P* < 0.001. The inter-observer ICC between observers A and B was 0.997 (95% CI 0.993–0.998), and *P* < 0.001. The results show that there is high consistency between the measured T_2_^*^ values within and between observers.

## Discussion

The primary treatment for most TM patients is supportive medical care. Patients with severe anemia require regular, lifelong blood transfusion and iron chelation therapy^20^. Advances in HSCT offer potential cure options for some patients. A recent case–control study noted that overall survival was similar in children and adults with TM regardless of HSCT or chronic transfusion therapy (82.6 ± 2.7%, 85.3 ± 2.7%, *P* = NS)^21^. However, HSCT is associated with improved health-related quality of life compared with chronic transfusion therapy^22–24^. Although the cost of treatment may vary by country, the estimated cost of HSCT is almost always lower than the cumulative cost of lifelong blood transfusion and conventional treatment with iron chelation therapy^25,26^. However, how does the level of organ iron load change before and after HSCT in TM patients?

In this study, the internal organ iron load of TM patients after HSCT was evaluated based on MRI T_2_^*^ technology, and it was found that there was no significant difference in L-T_2_^*^ and H-T_2_^*^ at 24 months after HSCT compared with that before HSCT. For patients more than 24 months after HSCT, L-T_2_^*^ and H-T_2_^*^ were significantly higher than those before HSCT, that is, the iron load of viscera was significantly reduced. This suggests that blood reconstitution in TM patients after HSCT is a slow process, which is the same view as Jagannath^4^. In the process of blood reconstitution, most TM patients still need transfusion therapy. In fact, some of the patients in this study still required regular transfusion therapy after HSCT. Regular blood transfusion is easy to cause iron load. Therefore, it is recommended that TM patients after HSCT should keep regular monitoring of organ iron load in the process of blood reconstitution (at least 24 months after HSCT), and make appropriate iron chelation therapy.

Hematopoietic stem cell transplantation replaces the ineffective erythropoiesis with an effective allogeneic substitute. The transplanted hematopoietic progenitor cells take over the function and synthesise normal red blood cells. The process enables to gradually correct anemia and eliminate the hemolytic process which improves the lifetime need for blood transfusion and chelation therapy^3^. However stem cells enter the bone marrow and, and within 2–4 weeks of transplantation, they begin to produce new white blood cells, red blood cells, and platelets^3,27^.

Blood smear of the first patient with TM undergoing HSCT showed typical changes in TM^27^, initial hemoglobin 5.9 g/dl, white ocyte count 3 × 10^9^/l, 50% nucleated red blood cells. Hemoglobin electrophoresis shows only the fetal haemoglobin. The hematocrit on days 29, 47, 97 day was 37%, 32%, 37%, respectively; white blood cell count was 0.66 × 10^9^/l, 3 × 10^9^/1, 3.4 × 10^9^/l, respectively. Liver and spleen enlargement completely disappeared by day 60. During the sixth month, the patient was well with a hemoglobin of 14.4 g/dl, white blood cell count 3.9 × 10^9^/l, a and fetal haemoglobin undetectable by electrophoresis. The whole process embodies the changing process of blood cells after HSCT in TM patients after HSCT, that is, it seems to take a longer time to show efficacy.

A retrospective analysis of 516 pediatric and adult thalassemia patients suggested that the 30-year survival rate after HSCT was similar to that expected for thalassemia patients receiving conventional care (transfusion, etc.), and that the vast majority of HSCT survivors were cured from thalassemia (94.2%)^21^. A large retrospective analysis by Baronciani et al. proposed a cure of 80–90% of thalassemia patients in actively addressing multiple post-HSCT factors, including improved pretreatment regimen, improved graft-versus-host disease prevention (GvHD), and more effective antimicrobial, antiviral and antifungal therapy. Overall, HSCT has a high cure rate for treating patients with thalassemia^28^.

Some studies have shown that there is no significant correlation between myocardial iron load and SF and liver iron load^29–32^. Chaosuwannakit^33^, Ghugre^34^ and Kaltwasser^35^ also showed that there was no significant correlation between myocardial iron load and SF, while there was a medium–low correlation between liver iron load and SF. The results of this study were similar, the overall L-T_2_^*^, H-T_2_^*^, and SF showed no or low correlation before and after HSCT in TM patients. High correlations in some subdivided subgroups are likely due to overfitting or sampling error resulting from the abrupt reduction in sample size after subgroup analysis. Obviously, the use of SF alone to monitor iron load in humans is not comprehensive, as this measure is likely to show considerable changes due to blood transfusion, inflammation, infection, or other chronic diseases. Some studies have also pointed out that SF may be too low when patients have severe iron deposition^34,36^. In conclusion, these results underscore the importance of T_2_^*^ MRI in assessing iron burden, especially in the liver and heart.

In this study, the statistics of red blood cell transfusion and iron removal therapy before HSCT in 115 TM patients were collected, and it was found that the red blood cell transfusion required by TM patients with different disease states was slightly different, 1.5–3 units of red blood cell transfusion accounted for a large proportion. All patients received iron chelation therapy with different methods, but because of the different medical compliance of patients in this region, there were 15 patients who did not receive regular iron chelation therapy. Different iron chelation regimens may affect the iron load of organs. However, more than 90% of the patients received regular iron chelation therapy, and we conducted subgroup analysis at different time points, and the results were reliable.

Limitations of this study: (1) Although 141 TM patients was included, the overall sample size was relatively adequate. However, when analyzing different subgroups, the sample size was relatively small. (2) As most of the TM patients in our center are β-TM, the number of α-TM patients included in our study is small, but it has little impact on the purpose of this study. (3) Due to the limited clinical data we collected, blood routine and liver and kidney function indexes were only presented as baseline data, without considering the main factors that may affect serum ferritin level. (4) This study only explored the changes of heart and liver iron load in TM patients before and after HSCT from the overall change trend, and did not control for the variable of iron chelators, that is, it can not prove that iron status/Mean change rates (%) are not touched by iron chelators, their doses, and amounts of blood transfusions. This point may become an entry point for the future work.

In conclusion, SF alone cannot effectively assess the change of iron load after HSCT. MRI T_2_^*^ technique is of great significance for quantitative assessment of cardiac and hepatic iron content in TM patients after HSCT. At least 24 months after HSCT, the heart and liver iron content of TM patients should be continuously and regularly monitored by SF and MRI, so as to better formulate the corresponding chelating iron removal plan.

## Data Availability

The datasets generated during and/or analysed during the current study are not publicly available, but are available from the corresponding author on reasonable request.
